# Comparison of serum amylase level between dipeptidyl peptidase-4 inhibitor and GLP-1 analog administration in patients with type 2 diabetes mellitus

**DOI:** 10.1186/s41043-019-0197-x

**Published:** 2019-11-14

**Authors:** Junichi Okada, Eijiro Yamada, Yawara Niijima, Shuichi Okada, Masanobu Yamada

**Affiliations:** 1 Department of Medicine and Molecular Science, Gunma University Graduate School of Medicine, 3-39-15 Showa-machi, Maebashi, Gunma 371-8511 Japan; 2 Kan-etsu Chuo Hospital, 71 Kitahara, Takasaki, Gunma 370-3513 Japan

## Abstract

We monitored serum amylase level in patients with type 2 diabetes mellitus (T2DM) prescribed either dipeptidyl peptidase-4 inhibitor or GLP-1 analog (GLP-1 group) as monotherapy. Patients were treated for a 36-month period. All subjects were non-smoker and did not take any alcoholic beverages. Forty-nine patients were prescribed DPP4is (DPP4i group), and 9 patients were prescribed GLP-1 analogs (GLP-1 group). The median of serum amylase levels in DPP4is group was 73 U/mL and the median of serum amylase levels in GLP-1 analog group was 76. Thus, there was no statistical significance between the two groups. However, the increased serum amylase levels in the three patients were observed only in the DPP4is group. One strength of the current study is that the serum amylase level was consistently measured in all subjects, and those subjects had been treated with either DPP4is or GLP-1 analogs as monotherapy. The incidence of elevated serum pancreatic amylase levels beyond normal range was calculated as 6.12% in the DPP4is group although the frequency was 0% in the GLP-1 analog group. Measurement of serum amylase consistently might have clinical meaning to catch the onset of pancreatitis and minimize the side effects due to DPP4is and GLP-1 analogs.

To the Editor:

Rathish et al. reported a significantly higher lipase level among the dipeptidyl peptidase-4 inhibitor (DPP4i) users in comparison with other oral hypoglycemic drug users [1]. On the other hand, whether DPP4is or GLP-1 analog users are associated with pancreatitis is not concluded yet [2, 3]. On the other hand, elevated serum amylase level is necessary for diagnosis of pancreatitis. Therefore, we consistently measured serum amylase levels in patients with type 2 diabetes mellitus (T2DM) before and after being prescribed either DPP4is or GLP-1 analogs as monotherapy.

Patients consistently visited our hospital for follow-up examination once a month, and blood glucose and HbA1c were measured from the same casual blood samples. In parallel, serum amylase level was measured every other month. Body weight and blood pressure were also measured each time. The present study includes patients that were followed up in this manner for a 36-month period. All subjects were non-smoker and did not take any alcoholic beverages. Blood samples for the relevant investigations were analyzed at our hospital. Procedures for measurement of the above investigations were well established and routinely done at the above laboratory [4].

Forty-nine patients were prescribed DPP4is (DPP4is group). The median age was 69 years (range 42~88 years). Sitagliptin was prescribed to 31 patients, vildagliptin was prescribed to 12 patients, linagliptin was prescribed to 4 patients, and anagliptin was prescribed to 2 patients. Nine patients were prescribed GLP-1 analogs (GLP-1 analogs group). The median age was 67 years (range 38~79 years). Dulaglutide was prescribed to 8 patients, and lixisenatide was prescribed to 1 patient. All of the patients did not suffer any type of pancreatic disease prior to start of either DPP4is of GLP-1 analogs.

We did not find statistically, significant difference about body weight, duration of diabetes mellitus, blood glucose, and HbA1c levels during the observation period between the DPP4is group and GLP-1 analog group. The median of serum amylase levels in the DPP4is group was 73 U/mL (range 33~209, reference range 49~136). The median of serum amylase levels in GLP-1 analogs group was 76 (range 48~120). There was no statistical significance between the two groups. However, three patients in DPP4is group showed transient elevation of serum amylase levels (157, 183, 209 respectively). Thus, the incidence of elevated serum pancreatic amylase levels beyond normal range was calculated as 6.12% in DPP4is group although the frequency was 0% in GLP-1 analog group. The increased serum amylase levels in those three patients were returned within normal range after the termination of DPP4is in less than 4 months. None of them showed any clinical symptom and abnormality in abdominal echo gram examinations.

One strength of the current study is that the serum amylase levels were consistently measured in all patients with T2DM in the current population every other month through the entire observation period. Measurement of serum amylase level consistently might have meaning to catch the onset of pancreatitis and minimize the side effects of DPP4is and GLP-1 analogs.

## Data Availability

Not applicable.

## Abbreviations

DPP4i: Dipeptidyl peptidase-4 inhibitor
